# Integrative approach to pedobarography and pelvis-trunk motion for knee osteoarthritis detection and exploration of non-radiographic rehabilitation monitoring

**DOI:** 10.3389/fbioe.2024.1401153

**Published:** 2024-07-31

**Authors:** Arnab Sarmah, Lipika Boruah, Satoshi Ito, Subramani Kanagaraj

**Affiliations:** ^1^ Department of Mechanical Engineering, Indian Institute of Technology Guwahati, Guwahati, India; ^2^ Graduate School of Engineering, Gifu University, Gifu, Japan; ^3^ Center for Intelligent Cyber Physical Systems, Indian Institute of Technology Guwahati, Guwahati, India; ^4^ Faculty of Engineering, Gifu University, Gifu, Japan

**Keywords:** knee osteoarthritis, rehabilitation, disease identification, pedobarography, wearable sensor, surface electromyography, non-radiographic

## Abstract

**Background:**

Osteoarthritis (OA) is a highly prevalent global musculoskeletal disorder, and knee OA (KOA) accounts for four-fifths of the cases worldwide. It is a degenerative disorder that greatly affects the quality of life. Thus, it is managed through different methods, such as weight loss, physical therapy, and knee arthroplasty. Physical therapy aims to strengthen the knee periarticular muscles to improve joint stability.

**Methods:**

Pedobarographic data and pelvis and trunk motion of 56 adults are recorded. Among them, 28 subjects were healthy, and 28 subjects were suffering from varying degrees of KOA. Age, sex, BMI, and the recorded variables are used together to identify subjects with KOA using machine learning (ML) models, namely, logistic regression, SVM, decision tree, and random forest. Surface electromyography (sEMG) signals are also recorded bilaterally from two muscles, the rectus femoris and biceps femoris caput longus, bilaterally during various activities for two healthy and six KOA subjects. Cluster analysis is then performed using the principal components obtained from time-series features, frequency features, and time–frequency features.

**Results:**

KOA is successfully identified using the pedobarographic data and the pelvis and trunk motion with the highest accuracy and sensitivity of 89.3% and 85.7%, respectively, using a decision tree classifier. In addition, sEMG data have been successfully used to cluster healthy subjects from KOA subjects, with wavelet analysis features providing the best performance for the standing activity under different conditions.

**Conclusion:**

KOA is detected using gait variables not directly related to the knee, such as pedobarographic measurements and pelvis and trunk motion captured by pedobarography mats and wearable sensors, respectively. KOA subjects are also distinguished from healthy individuals through clustering analysis using sEMG data from knee periarticular muscles during walking and standing. Gait data and sEMG complement each other, aiding in KOA identification and rehabilitation monitoring. It is important because wearable sensors simplify data collection, require minimal sample preparation, and offer a non-radiographic, safe method suitable for both laboratory and real-world scenarios. The decision tree classifier, trained with stratified k-fold cross validation (SKCV) data, is observed to be the best for KOA identification using gait data.

## 1 Introduction

Osteoarthritis (OA) is the most prevalent type of musculoskeletal disorder globally and is the leading cause of chronic pain and disability in adults (Singh et al., 2022). Knee osteoarthritis (KOA) accounts for four-fifths of the burden of OA worldwide. The pooled global prevalence of KOA is 16% in individuals aged 15 and over and 22.9% in individuals 40 and over. The ratio of prevalence and incidence in women and men is found to be 1.69 and 1.39, respectively (Cui et al., 2020). In India, the prevalence is reported to be 28.7%. The prevalence is higher in women at 31.6% than in men at 28.1% (Pal et al., 2016). KOA is a degenerative disorder and requires total knee replacement, i.e., knee arthroplasty at an advanced stage of the disease. It, however, results in substantial health costs. Thus, an important aspect of managing the disease is early identification and, hence, early intervention (Cui et al., 2020). The diagnosis of KOA can be confirmed based on clinical and/or radiological features. The current gold standard for diagnosing OA is X-ray imaging, which is cost-efficient and widely available. However, it is insensitive to detecting early OA changes (Tiulpin et al., 2018) and involves high-energy electromagnetic radiation. MRI has also been increasingly employed to diagnose KOA. However, it can detect OA with high specificity and moderate sensitivity. Thus, it is more useful for ruling out OA than ruling it in (Menashe et al., 2012). Thus, early diagnosis of OA is particularly challenging as it relies heavily on the subjective judgment of the practitioner due to the lack of a precise grading system. The widely employed Kellgren–Lawrence (KL) grading scale is semi-quantitative and suffers from ambiguity. Such ambiguity poses an obstacle to early OA diagnosis, thus affecting millions of people globally (Tiulpin et al., 2018). Machine learning (ML) has been employed for the diagnosis of KOA using kinematics (Yang et al., 2020; Kwon et al., 2019; Kwon et al., 2020) and kinetics (Kwon et al., 2019) of the hip, knee, and ankle joints. They have also been employed along with radiographic images (Kwon et al., 2020) to identify KOA subjects from healthy subjects (Yang et al., 2020) and differentiate between the different grades of KOA subjects (Kwon et al., 2019; Kwon et al., 2020). However, the data are collected using either a 3D motion capture system (Kwon et al., 2019; Kwon et al., 2020) or multiple IMUs (Yang et al., 2020), which require a post-processing step before the data can be used for classification. In addition, classification is performed using only one type of classifier. Surface electromyography (sEMG) has also been employed in recent years for the diagnosis of KOA, with a high accuracy of 92% (Chen et al., 2019) and 96.3% (Khader et al., 2024). Data considered for diagnosis were collected considering walking at a self-selected pace as an activity. The different muscles considered are the quadriceps femoris (Khader et al., 2024), medial gastrocnemius (Khader et al., 2024), rectus femoris (Khader et al., 2024), semi-tendinous (Chen et al., 2019; Khader et al., 2024), biceps femoris (Chen et al., 2019; Khader et al., 2024), and vastus lateralis (Chen et al., 2019; Khader et al., 2024).

The diagnosis is followed by an intervention regimen, which revolves around a combination of non-pharmacological and pharmacological methods. One of the initial measures is weight reduction, which can help slow down the progression of KOA. Another most widely implemented remedy is physical therapy and rehabilitation. It has been useful for patients with pain and mobility. Specific useful programs include strength training, Tai Chi, aerobics, electrotherapy, and hydrotherapy, among which strength training is the most common approach. It improves the muscular strength and joint stability of the individual, thus improving Western Ontario and McMaster Universities (WOMAC) pain scores and overall health benefits (Bhatia et al., 2013). Increased rectus femoris muscle force is related to thinner knee joint cartilage in KOA (Yagi et al., 2022), and increased muscle activations of the biceps femoris have been reported in KOA subjects compared to healthy subjects while performing activities of daily life (Hortobágyi et al., 2005). Thus, the strength of the contraction of periarticular muscles (i.e., the quadriceps and hamstrings for the knee joint) is an important contributing factor to the quality of the cartilage. In addition to increasing the strength of the muscles surrounding the knee, it also increases the intra- and intermuscular coordination of the knee extensor muscles, which results in lower impact and impulsive loading being transmitted through the joint (Beckwée et al., 2013). The assessment of the effectiveness of the physical therapy further enables us to plan the intervention regimen and understand the progress. One of the approaches includes the use of the QQ index (Brouwer et al., 1999), where the subjects’ effective working hours during a day are compared to those of the previous day (Reijonsaari et al., 2012). Another approach is through a video system or a mobile application through which the physiotherapist can remotely monitor a patient in real time and provide instant feedback (Saraee et al., 2017; Vaish et al., 2017)).

Since the assessment of the effectiveness of physical therapy for KOA is subjective and relies on the clinician’s expertise, employing a quantitative approach becomes highly beneficial in achieving more objective results. The effectiveness of physical therapy on KOA has been reported to be monitored in a case study that showed improvements in temporal parameters such as stride length, mean velocity, and cadence. Root mean square (RMS) values of EMG are also used to infer the improvement of the condition post-treatment (Liang et al., 2019). Physical therapy has also been monitored using a pedometer, Fitbit, and accelerometer to assess the influence of physical activity on a wide range of subjects with different pathologies (de Leeuwerk et al., 2022). However, it only utilizes kinematic variables and does not consider the kinetic variables associated with human movement. Thus, there is a need for the identification of kinetic and kinematic variables that can be used for KOA diagnosis and also for monitoring the effectiveness of any intervention.

The highly interdependent nature of human movement allows us to employ variables associated with other joints and muscles to assess their effect on other parts and *vice versa*. It has been reported that the quadriceps and hamstring muscles weaken and have delayed reaction time in subjects suffering from plantar fasciitis (Lee et al., 2020). Increased hamstring tightness also induces prolonged forefoot loading (Harty et al., 2005). In addition, hamstring length significantly influences the pelvic angle and flexion range of motion (ROM), lumbar angle flexion ROM, and thoracic angle flexion ROM. Short hamstrings are associated with decreased flexion ROMs of the pelvic and lumbar angles and increased flexion ROM of the thoracic angle (Gajdosik et al., 1994). This shows that there exists a relationship between the muscular strength of the periarticular muscles of the knee, the plantar pressure, and the ROM of the pelvis and lumbar region.

This work thus aims to identify KOA subjects using both kinetic and kinematic non-knee joint parameters, which are the pedobarographic measurements and the pelvis and trunk ROM variables. In addition, the effect of KOA on the periarticular muscles of the knee is studied through sEMG, and using these data, the kinetic and kinematic variables are to be established as potential biomarkers for monitoring the effectiveness of any neuromuscular rehabilitation intervention technique for addressing KOA. This is possible as knee health has been associated with hamstring and quadriceps muscle strength and is also associated with pelvis and trunk ROM along with plantar pressure distribution. The variables considered for KOA identification are dynamic in nature and require minimal subject preparation.

## 2 Methodology

**FIGURE 1:**
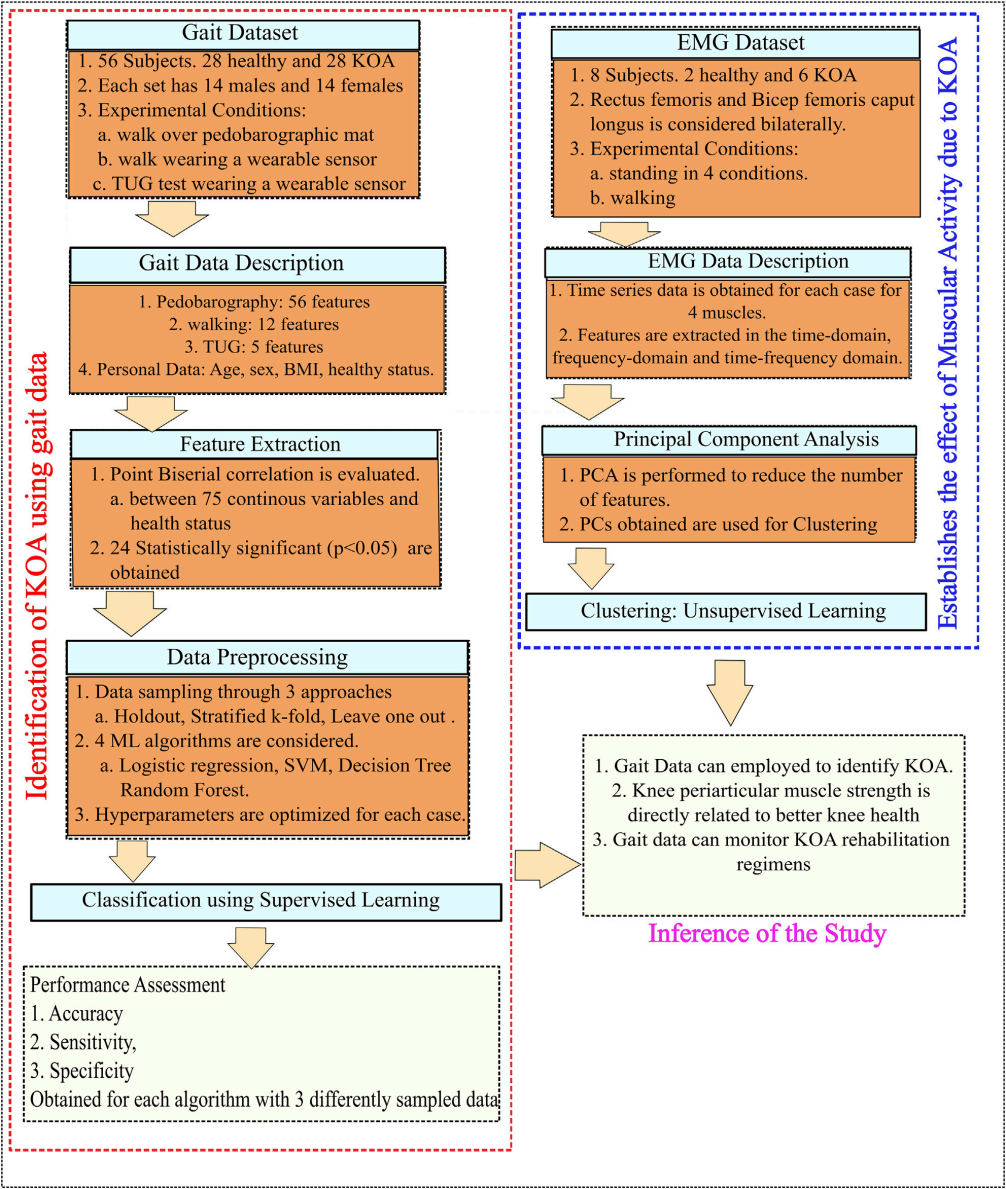
Flowchart of the methodology.

Figure 1 shows us an overview of the various steps undertaken during the study. It includes a two-fold methodology to investigate KOA identification and monitoring through gait and muscle data. Initially, gait data from 56 subjects (28 healthy and 28 KOA) are analyzed using supervised ML algorithms to demonstrate the ability to identify KOA using beyond knee-related gait data. The effect of KOA on the periarticular muscles of the knee is then studied through the analysis of sEMG data from the rectus femoris and bicep femoris caput longus bilaterally. The features obtained are then used for cluster analysis. Successful clustering will indicate the effect of KOA on the muscular activity of the periarticular muscles. Integrating the two studies, we can propose using gait data to monitor muscle strengthening to rehabilitate KOA subjects. Data are collected using the wearable sensor, pedobarographic mat, and sEMG sensors. Pelvis ROM is captured using the wearable sensors while the subjects walk comfortably at a self-selected speed for 20 m. The wearable sensor was also used to conduct the Timed Up and Go (TUG), which provides the trunk ROM in the sagittal plane during the sit-to-stand and stand-to-sit parts of TUG and the total TUG time. TUG is considered because it is recommended by the Osteoarthritis Research Society International (OARSI) as an assessment tool in KOA, which is necessary to detect functional mobility and the risk of falls. Measuring trunk movement during these steps helps health workers and physiotherapists provide a proper rehabilitation strategy for KOA (Dobson et al., 2013). The subjects walk at a self-selected speed over the pedobarography mat to capture the 56 kinetic and kinematic features. There is evidence of neuromuscular adaptations associated with even early stages of KOA and without gait adaptations (Duffell et al., 2014). Thus, sEMG data are collected from two muscles, namely, the rectus femoris and the biceps femoris caput longus, while the subjects walk at a self-selected speed. sEMG data are also collected while standing under different stability conditions. Walking is chosen because it has been reported that individuals with KOA exhibit higher gait deviations than healthy subjects (Mills et al., 2013). KOA has also been reported to cause deficits in balance control, with its severity increasing in moderate to severe KOA (Kim et al., 2011). In addition, standing data under different stability conditions have been reported to be useful in classifying balance-related disorders (Sarmah et al., 2024). Hence, standing under different stability conditions are considered for sEMG data collection.

### 2.1 Participants

The study was approved by the Institute Human Ethics Committee (IHEC), IIT Guwahati, and conducted in compliance with the relevant regulations. Written informed consent is obtained from all the study participants. Pedobarography and wearable sensor data from the pelvis are collected from KOA subjects with different degrees of severity, as well as from healthy subjects. Age, sex, and BMI are recorded for each subject and are summarized in Table 1. Data are collected for 28 able bodied subjects and 28 subjects with KOA. The inclusion criteria for healthy subjects are (i) age greater than 18 years and (ii) ability to perform normal activities of daily life. For KOA subjects, the inclusion criteria are (i) age greater than 18 years, (ii) being diagnosed with KOA and referred by an orthopedic doctor, and (iii) being able to walk and stand without the need for any support. The exclusion criteria in both healthy and KOA cases are (i) diagnosed with neurological disorders like Parkinson’s disorder. The 28 KOA subjects contain 11 subjects with Grade 1 severity, 13 subjects with Grade 2 severity, and 4 subjects with Grade 3 severity according to KL grade.

**TABLE 1:** Summary of the subject characteristics in two categories.

	Age	Sex	BMI
Healthy subjects	30.3 ± 7.8	14 male and 14 female subjects	24.1 ± 3.8
KOA subjects	53.8 ± 11.7	14 male and 14 female subjects	27.5 ± 4.3

### 2.2 Gait data collection

**FIGURE 2:**
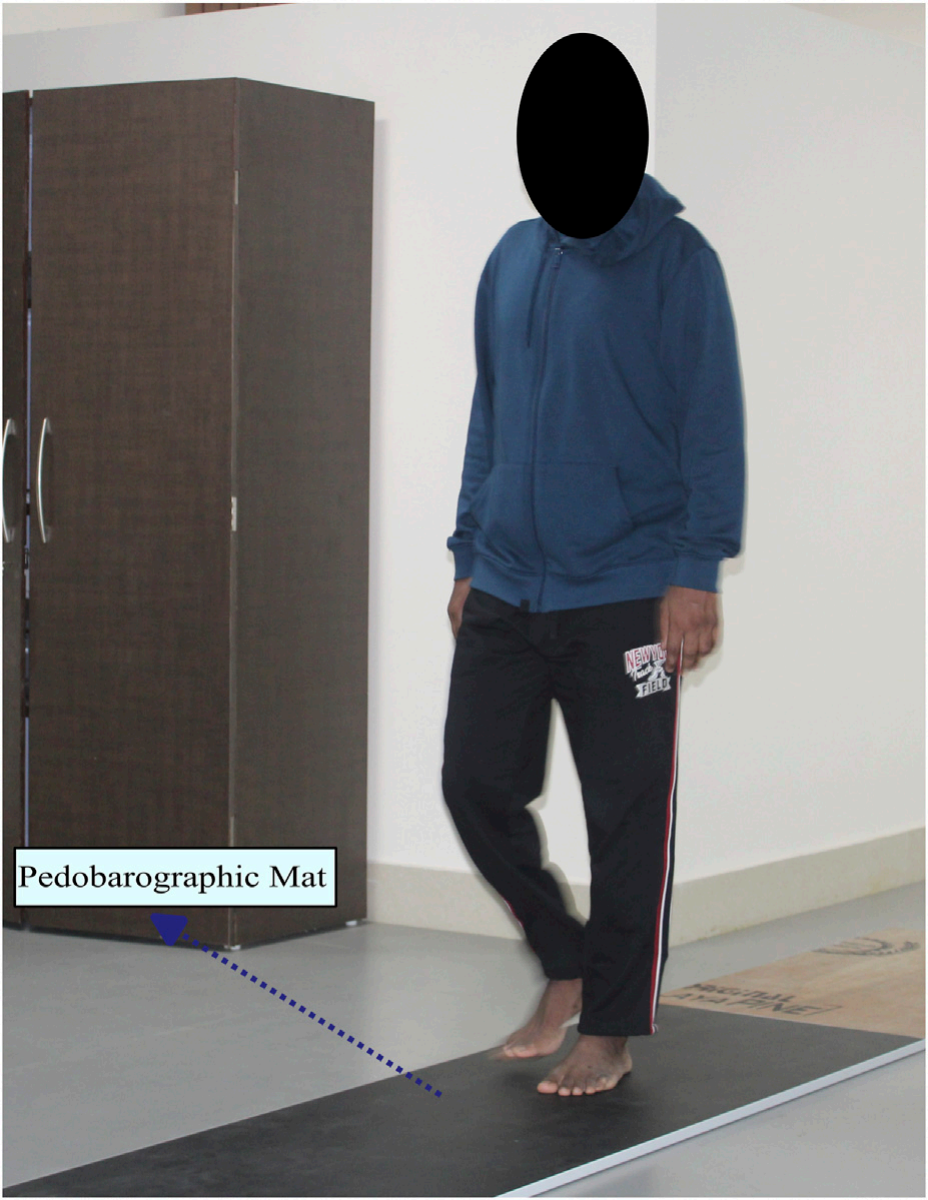
Sample subject walking over the pedobarographic mat.

All the gait data are collected in the Gait and Motion Analysis Laboratory at IIT Guwahati. The subjects are, at first, familiarized with the experimental setup and protocol. They are instructed to walk over a dynamic pedobarographic mat from Zebris Medical GmbH (FDM-2) at a self-selected speed, as shown in Figure 2. This mat captures the plantar pressure experienced by the subject during walking, along with the spatiotemporal variables such as stride length, step length, cadence, and speed.

**FIGURE 3:**
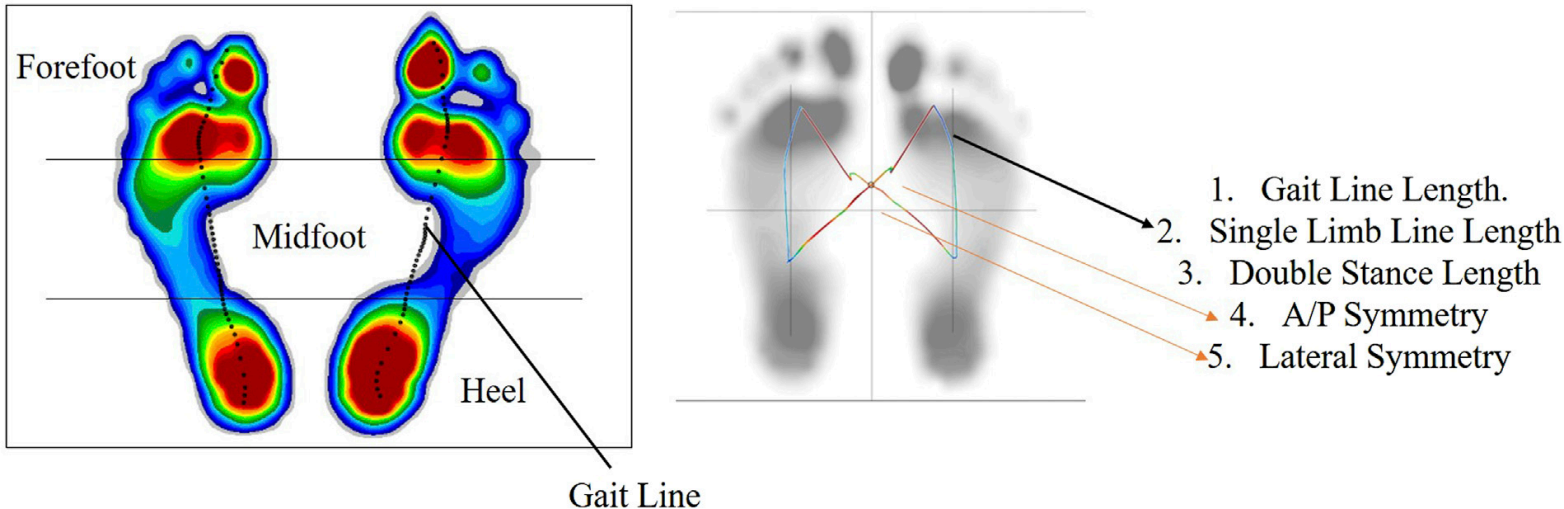
Representation of the dynamic variables obtained from the pedobarographic mat.

**FIGURE 4:**
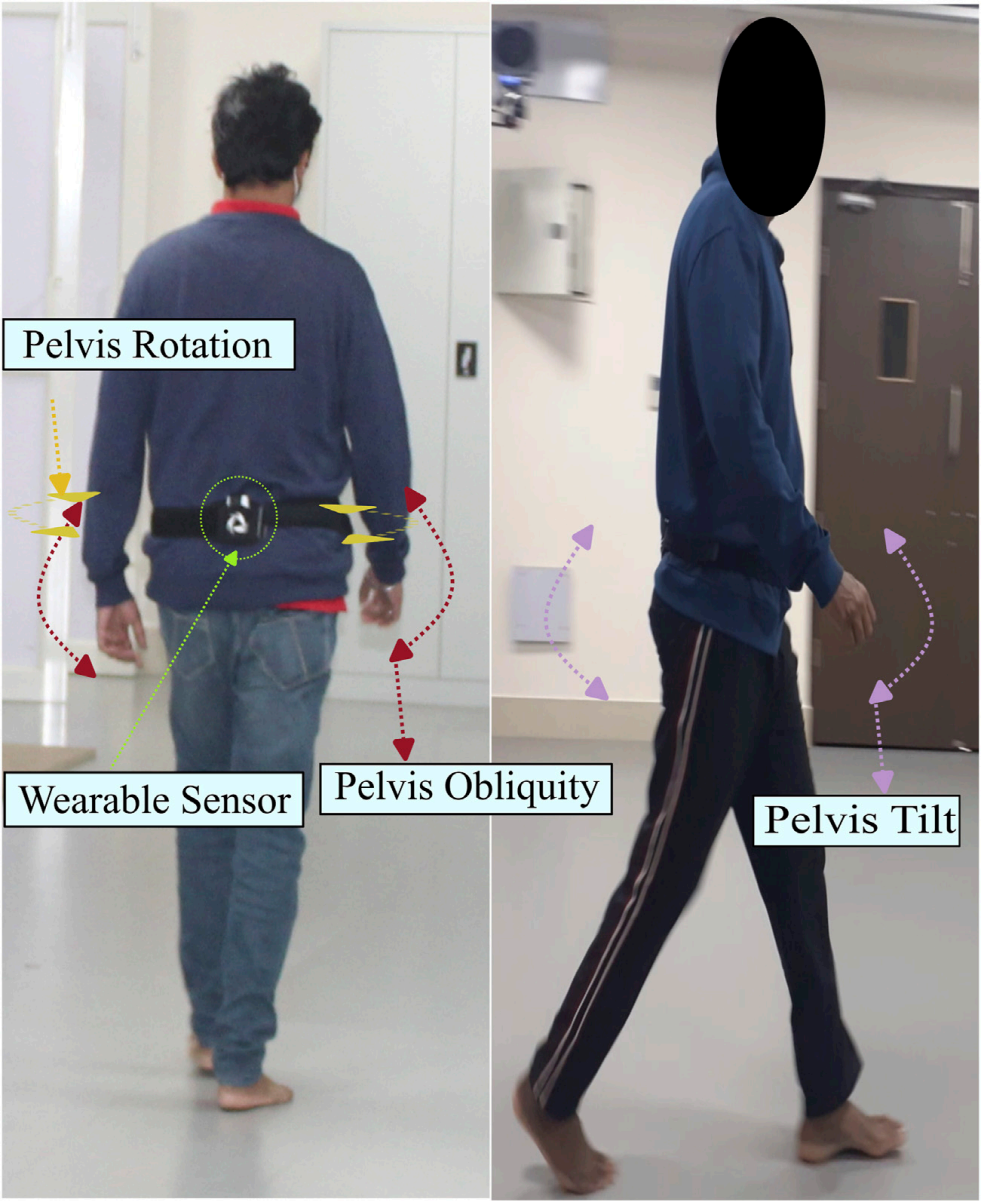
Pelvis motion in three planes during normal walking.

The dynamic variables captured include the force and pressure experienced in the three sections of the foot, namely, the forefoot, midfoot, and heel. In addition, butterfly parameters such as gait line length and velocity, single-stance line, anterior–posterior position, and mediolateral position are reported. A representation of the dynamic variables is shown in Figure 3. Thereafter, data are collected using the wearable sensor GWALK from BTS Bioengineering for two activities: normal walking and TUG. In the normal walk test, the wearable sensor is placed on the level of the subject’s sacrum (Supplementary Material S1) and then asked to walk at a self-selected speed for a distance of 20 m. Figure 4 shows the placement of the wearable sensor and the representation of the pelvis motion in the three planes. The variables captured during the normal walking test are pelvis tilt right (PTR), pelvis tilt left (PTL), pelvis obliquity right (POR), pelvis obliquity left (POL), pelvis rotation right (PRR), and pelvis rotation left (PRL) with respect to gait cycle percent. During the measurement of plantar pressure and pelvis ROM, the experimental condition is walking at a self-selected speed. However, ROM was not measured while the participant was walking over the foot pressure mat. It is because the GWALK requires the subject to walk at least 7 m, while the foot pressure mat is 2 m in length.

**FIGURE 5:**
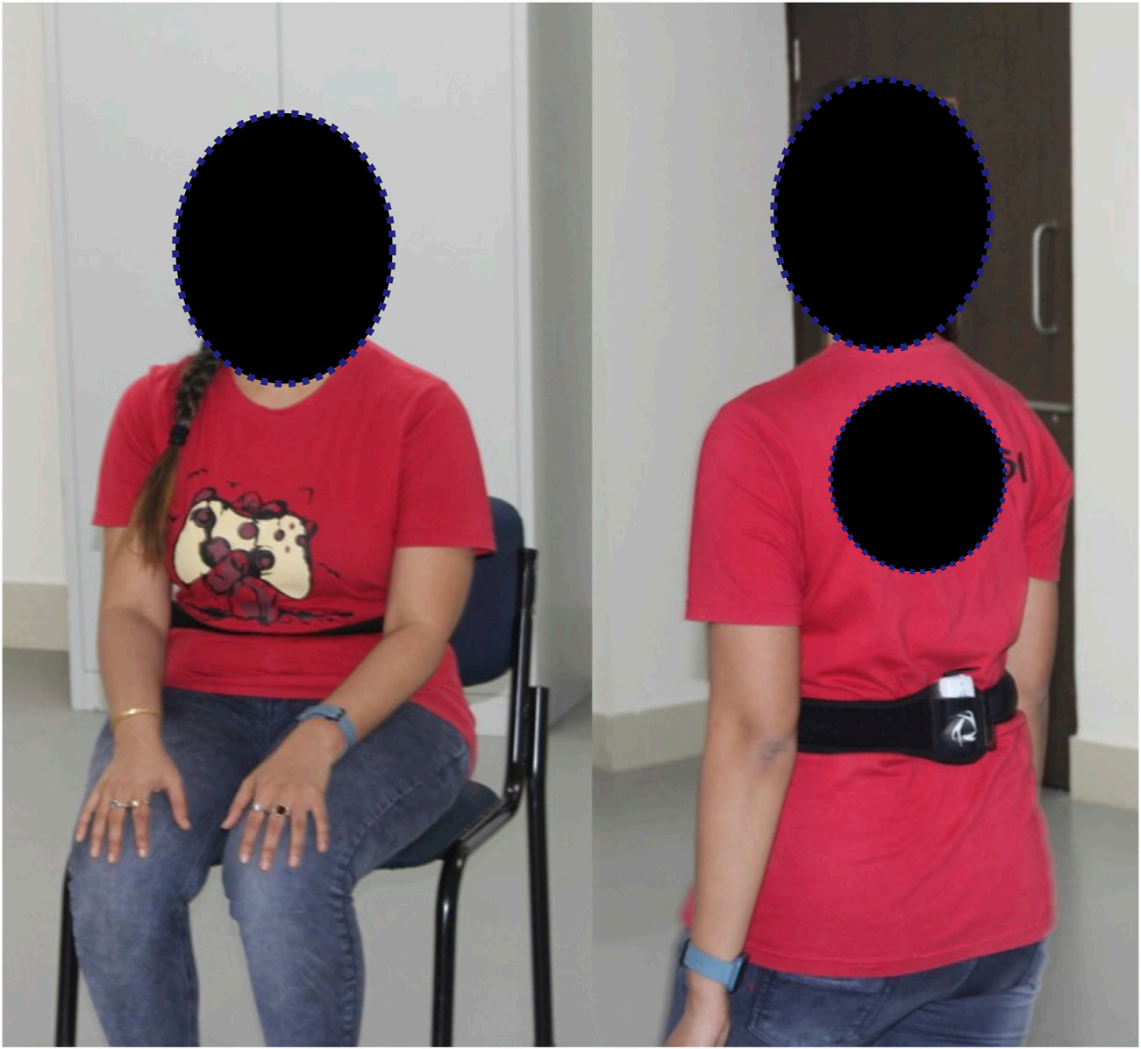
Sample subject during the TUG test.

In the TUG test, the wearable sensor was placed on the lumbar 2 (L2) vertebrae. The test starts with the subject in a seated posture, getting up and walking for 3 m, then turning around, and returning to the starting seated posture. A subject undergoing the test is shown in Figure 5. The variables captured from this experiment are the trunk flexion-extension (F/E) in the sagittal plane with respect to normalized time and the TUG time.

## 3 Data analysis

### 3.1 Data description

The data analysis and classification were performed using Python 3.11. Pelvis angles are obtained with respect to the gait cycle percent, and the mean and standard deviation (SD) are evaluated for each subject under six conditions (considering the cardinal planes and the side of the body) using Eqs 1, 2. A total of 12 features, namely, the mean and SD of PTR, PTL, POR, POL, PRR, and PRL, are evaluated, which represent the pelvis angles in three planes during a normal walk. The mean and standard deviation (SD) are also evaluated for the trunk F/E angles in sit-to-stand and stand-to-sit conditions using Eqs 1, 2. Four trunk motion features, two mean and two SD values, which represent the trunk F/E during sit-to-stand and stand-to-sit activity, and TUG time are obtained from the TUG test. The mean and SD of different gait variables are considered as they have been successfully employed as a feature to classify between healthy and neuromusculoskeletal disorders, which result in gait deviation (Xia et al., 2015; Nandy, 2019). In addition, this approach reduces the computational complexity of the analysis due to the reduced dataset dimensionality.

$${Angle}_{mean}=\sum \left({Angle}_{i}\right)/N,$$ (1)

$${Angle}_{stddev}=\sqrt{\sum {\left({Angle}_{i}-{Angle}_{mean}\right)}^{2}/N-1,}$$ (2)

where Angle_i_ is the angle (pelvis or trunk) at each interval and N = 100 (gait cycle percentage).

In addition, 56 features are obtained from the pedobarographic mat, which includes 19 spatiotemporal features, 13 butterfly features, and 24 dynamic features. The spatiotemporal variables include the mean and SD of the foot progression angle (left and right side), step length (left and right side), stride length, mean and SD of step width, stance phase percent (left and right side), swing phase percent (left and right side), double stance phase percent, step time (left and right side), stride time, cadence, and velocity. The Foot progression angle is considered because gait training with a specific foot progression angle increases the lateral knee muscle co-activity, thereby unloading the medial knee compartment (Gholami et al., 2022). This implies that any change in knee joint loading will be reflected in the foot progression angles. The butterfly parameters consist of the mean and SD of the gait line length (left and right side), single limb support line (left and right side), anterior–posterior (A/P) position, and lateral symmetry and the maximum gait line velocity. The dynamic parameters obtained are the mean and SD of the forefoot, midfoot, and heel force and pressure (left and right side).

### 3.2 Feature selection using point-biserial correlation

Two personal features (age and BMI), 56 features from the pedobarographic data (19 spatiotemporal features, 13 butterfly features, and 24 dynamic features), 12 features from the Pelvis motion during normal walk, four features from the trunk movement during TUG, and TUG time are considered for analysis. At first, point-biserial correlation is evaluated between the 75 features and the health status of the subject by considering healthy as 0 and KOA as 1. Point-biserial correlation is performed when one of the variables is continuous and the other is a dichotomous variable. It is performed to find the variables strongly affected by KOA. The correlation coefficients obtained give us variables that are most affected by KOA. A total of 24 statistically significant (*p* < 0.05) variables that relate the variables to the health status of the subject are obtained from the point-biserial correlation, which can then be employed for the identification of subjects with KOA. The variables obtained after point-biserial correlation are shown in Table 2 in descending order of correlation coefficient. A total of 10 of the features are positively correlated with KOA, and 14 are negatively correlated. Age, SD of trunk F/E (sit to stand), and mean of trunk F/E (stand to sit) are the most positively correlated variables; and velocity, step length (left), and stride length are the most negatively correlated variables employed for classification.

**TABLE 2:** Point-biserial correlation relating the variables with the health status of the subjects.

Sl. No.	Variable	Point-biserial correlation coefficient	*p*-value
1	Age	0.708968	9.68E-10
2	Trunk F/E (sit-to-stand) SD	0.411328	0.001636
3	Trunk F/E (stand-to-sit) mean	0.343412	0.009564
4	TUG time	0.318584	0.016711
5	Double-stance phase %	0.314555	0.018221
6	Stance phase (right)%	0.29898	0.025197
7	BMI	0.281456	0.035606
8	Stance phase (left)%	0.27304	0.041748
9	Stride time (sec)	0.272102	0.042484
10	Gait line right SD	0.26594	0.047585
11	Swing phase (left) %	−0.27304	0.041748
12	Cadence (steps/min)	−0.28389	0.033977
13	Swing phase (right) %	−0.29898	0.025197
14	Single-limb support line right (mm)	−0.30409	0.022695
15	Heel left (pressure)	−0.30774	0.021037
16	PRL SD	−0.32316	0.015126
17	PRR SD	−0.3259	0.014239
18	Heel right (pressure)	−0.33572	0.011422
19	Single limb support line left (mm)	−0.34601	0.008998
20	Gait line right	−0.34897	0.008388
21	Step length right (cm)	−0.41402	0.001514
22	Stride length (cm)	−0.4395	0.000702
23	Step length left (cm)	−0.44234	0.000641
24	Velocity (km/hr)	−0.45057	0.000493

### 3.3 Data preprocessing

The 24 statistically significant variables, obtained after point-biserial correlation, include two personal features (age and BMI), 17 features from the pedobarographic data (11 spatiotemporal features, 4 butterfly features, and 2 dynamic features), 2 features from the pelvis motion during a normal walk, and 2 features from the trunk movement during TUG and TUG time. The statistically significant variables, sex, and pathological condition of the subject are used to identify subjects with KOA.

Data preprocessing is to be conducted before it can be used for identification. When applying a classification model, it is crucial to convert the dataset into numerical form. The pathology of the subject is already assigned in binary form. Binary data are then extracted from the other categorical variables, which results in an increase in the number of unique features such as sex, which gets split into two independent features, female and male. The “sex” column and one of the independent features are then dropped. In this case, the “female” column was dropped. In the “male” column, 0 indicates female subjects and 1 indicates male subjects. This method is called one-hot encoding. The “StandardScaler” function from Python’s scikit-learn library is then employed to normalize the data, ensuring a mean of 0 and a standard deviation of 1.

The data sampling for training and testing uses three approaches, namely, holdout method, stratified k-fold cross-validation (SKCV) method, and leave-one-out cross-validation (LOOCV) method. In the holdout method, the dataset is divided into training and test sets, which are to be used for training and testing, respectively. In this case, training and test sets comprise 75% and 25% of the total data, respectively. Stratified k-fold splits the dataset randomly into ‘k’ groups while ensuring that each fold has the same proportion of the different classes as the entire dataset. The models are then trained on the training set and analyzed on the testing set. The process is repeated k-times until each set/fold has been utilized as a test set. The data are divided into “5” folds or groups in this case. In the leave-one-out approach, each observation is considered the test set, and the remaining (N-1) observations are considered the training set. The process is repeated N times until each observation has been used as the testing set. Different types of sampling are employed to make the trained and tested models applicable to classify data from several different datasets, which may be unbalanced. Different sampling methods are utilized for the analysis to ensure the quality and reliability of the models. The stratified k-fold approach provides more reliable performance estimates and is crucial for imbalanced datasets. The leave-one-out approach is suitable for small datasets and assesses the model’s stability.

### 3.4 Hyperparameter optimization

Hyperparameters serve as crucial external configuration variables in managing machine learning models and are set before the model’s training. The process of finding the right set of hyperparameters is known as hyperparameter tuning or optimization. It involves experimenting with different combinations to maximize or minimize a target variable, often accuracy. Among the approaches employed, grid search stands out, systematically exploring all possible hyperparameter combinations from a predefined list to find the best fit. The optimization process aims to enhance the model’s performance on unseen data, thereby enhancing its overall predictive accuracy. The grid search approach is employed for hyperparameter search in all the models.

In logistic regression, different combinations of regularization parameters (which control the bias-variance trade-off to develop more generalized models), penalty terms, and the maximum number of iterations are scrutinized to optimize the logistic regression model’s performance. The tuning process optimizes the logistic regression model and makes it more generalized and resistant to overfitting. The optimized model is then trained and tested on three differently sampled datasets and provides insight into its effectiveness across various validation scenarios.

In the support vector machine (SVM), the different combinations of regularization parameters, kernel types (linear, polynomial, radial bias function, and sigmoid), degree (only for polynomial kernels), and gamma (only for polynomial, radial bias function, and sigmoid kernels) are explored. The choice of the optimal kernel depends on the dataset’s characteristics, such as linearity or non-linearity. The best kernel type selected might differ between the sampling methods (holdout, stratified K-fold, and leave one out) due to the distinct subsets they provide for training and testing, influencing the hyperparameter selection.

In the case of the decision tree classifier, key hyperparameters such as maximum depth (which is a limit to stop further splitting of nodes when the specified tree depth is reached), criterion for data splitting, cost complexity pruning (which addresses the problem of overfitting by selectively removing certain parts of the decision tree), minimum sample leaf (the minimum number of samples required for a leaf node or external node and hence do not have any further splits), and minimum sample split (the minimum number of samples required to split an internal split) are adjusted to find the combination that maximizes the model’s performance. These adjustments enhance the model’s performance by finding the right combination that maximizes accuracy.

In a random forest, the key hyperparameters include the number of estimators, which represents the number of trees in the random forest, maximum depth, minimum sample leaf, and minimum sample split. Collectively, these hyperparameters shape the structure and complexity of each decision tree within the random forest ensemble.

### 3.5 Classification

Eleven spatiotemporal variables, four butterfly features, and two dynamic features from pedobarography, namely, double stance phase %, right and left stance phase %, stride time, right and left swing phase %, cadence, right and left step length, stride length, velocity, mean and SD of right gait line, right and left single limb support line, right and left heel pressure along with two features from normal walking pelvis motion, namely, SD of PRL and PRR; two trunk motion features, namely, mean of trunk F/E during the stand-to-sit condition and SD of trunk F/E during the sit-to-stand condition and TUG time during TUG test, and personal details including BMI, age, sex, and the health status of the subject are used to identify subjects with KOA. The models considered for classification are logistic regression, SVM, decision tree, and random forest. These models are trained and tested on the three differently sampled datasets. Logistic regression explains the relationship between the dependent variable, i.e., the pathology of the subject, and the remaining independent input variables, i.e., the pedobarographic data, pelvis and trunk motion, and personal details, to classify subjects with KOA. The threshold for classification is considered to be ≥ 0.5. The optimized hyperparameter values for logistic regression are shown in Table 3.

**TABLE 3:** Hyperparameters obtained for logistic regression.

Hyperparameter	Holdout-sampled data	Stratified K-fold-sampled data (K = 5)	Leave-one-out-sampled data
Regularization parameter (C)	0.1	10	10
Penalty	L2	L2	L2
Maximum number of iterations	100	100	100

SVM classifies the data points by finding a hyperplane in an N-dimensional space. The “best” hyperplane is chosen among the several hyperplanes developed. The optimized hyperparameters for SVM are shown in Table 4. After determining the ‘optimal’ hyperplane, the data points situated on either side of it are assigned to distinct classes. The classification of an unknown data point is then based on its relative position to this established hyperplane. SVM with a linear kernel is used for the holdout- and stratified k-mean-sampled data and SVM with a radial bias function (rbf) kernel is employed for leave-one-out-sampled data.

**TABLE 4:** Hyperparameters obtained for SVM.

Hyperparameter	Holdout-sampled data	Stratified K-fold-sampled data (K = 5)	Leave-one-out-sampled data
Regularization parameter (C)	1	1	10
Kernel	Linear	Linear	rbf
Degree	Not applicable	Not applicable	Not applicable
Gamma	Not applicable	Not applicable	0.01

A decision tree is constructed as a flowchart-like tree structure and employs internal nodes to test different attributes; branches represent the results of those tests, and each leaf node represents the class the feature falls into. The decision tree is built through a recursive process that involves dividing the training data into subsets according to attribute values. This recursive splitting continues until a predefined stopping criterion is satisfied, such as reaching the maximum tree depth or fulfilling the minimum number of samples needed to split a node. The decision tree is constructed by recursively splitting training data into subsets based on the values of the attributes until a stopping criterion is met, such as the maximum depth of the tree or the minimum number of samples required to split a node. During the training process, the algorithm selects the optimal attribute for data splitting using metrics such as entropy or gini impurity, aiming to maximize information gain or minimize impurity after each split. To achieve the best results, hyperparameters, namely, the maximum depth, criterion, cost complexity pruning, minimum sample leaf, and minimum sample split, are optimized. The hyperparameters obtained for the different sampled data for the decision tree are listed in Table 5.

**TABLE 5:** Hyperparameters obtained for decision tree.

Hyperparameter	Holdout-sampled data	Stratified K-fold-sampled data (K = 5)	Leave-one-out-sampled data
Maximum depth	7	7	3
Criterion	Entropy	Gini	Entropy
Cost complexity pruning	0.05	0	0.025
Minimum sample leaf	1	3	1
Minimum sample split	4	6	2

A random forest classifier is an ensemble method where a collection of decision trees is trained on different subsets of the data. It employs the “bagging” approach for ensemble, in which each tree trains a random subset of the dataset, sampled with replacement. The final classification is determined by a majority vote among the individual trees. The different hyperparameters associated with the random forest classifier are the number of trees, minimum sample leaf, and minimum sample split. The hyperparameters obtained for the different sampled data for the random forest are listed in Table 6.

**TABLE 6:** Hyperparameters obtained for random forest.

Hyperparameter	Holdout-sampled data	Stratified K-fold-sampled data (K = 5)	Leave-one-out-sampled data
Number of trees	100	100	50
Minimum sample leaf	1	1	1
Minimum sample split	2	5	2

### 3.6 Performance assessment

Accuracy, sensitivity, and specificity metrics are used to assess the performance of the classifiers. True positive (TP) signifies the correct identification of KOA (positive) cases, while true negative (TN) indicates the accurate exclusion of healthy (negative) cases. False positive (FP) occurs when the classifier wrongly identifies a negative (healthy) case as positive (KOA), and false negative (FN) arises when a positive (KOA) case is incorrectly classified as negative (healthy). The overall accuracy is computed by (TP + TN)/TCT, where TCT represents the total number of classification tests. Sensitivity and specificity are expressed by TP/(TP + FN) and TN/(TN + FP), respectively. Accuracy provides an overall measure of model performance, sensitivity gauges the model’s ability to detect positive cases, and specificity assesses the accuracy of identifying negative outcomes. Evaluation is conducted across three datasets sampled using holdout, SKCV, and leave-one-out LOOCV approaches.

### 3.7 Results

**FIGURE 6:**
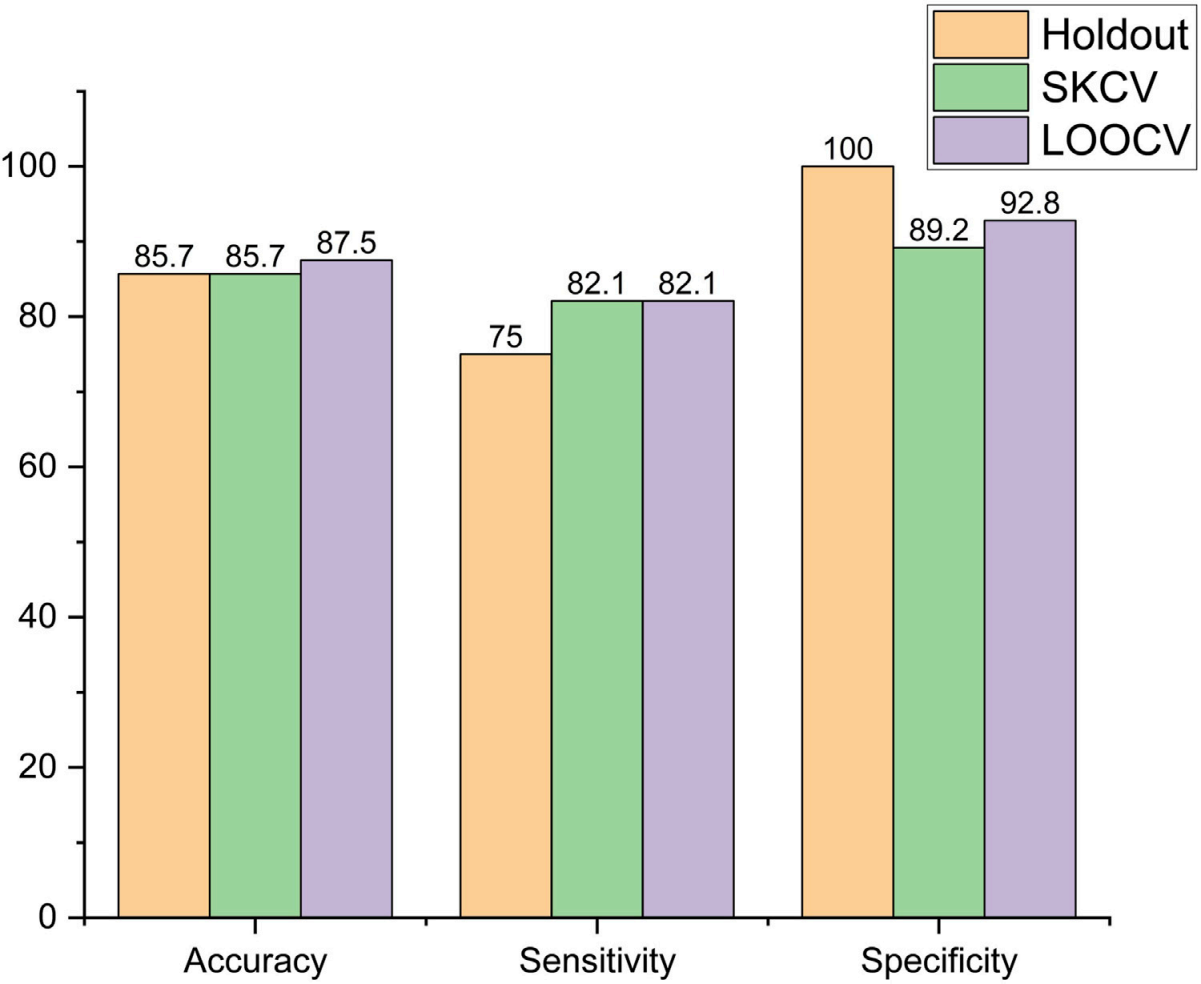
Performance metric of logistic regression.

**FIGURE 7:**
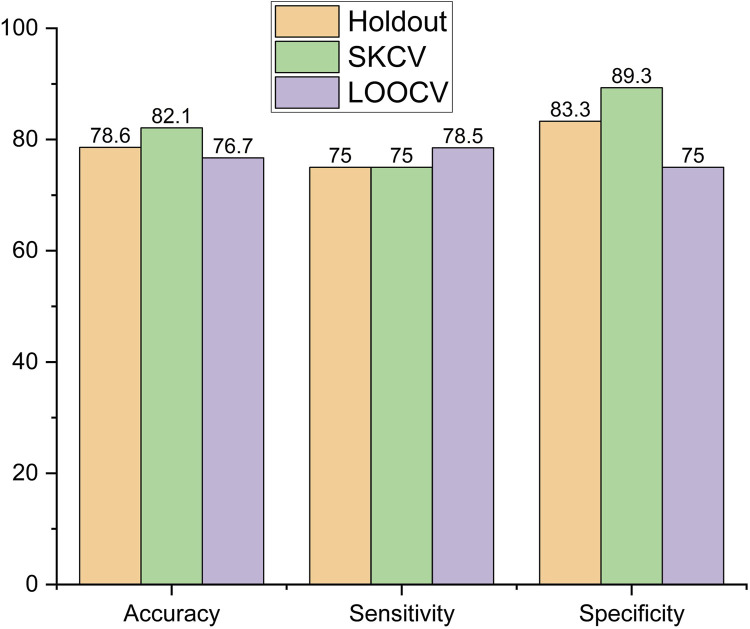
Performance metric of SVM.

**FIGURE 8:**
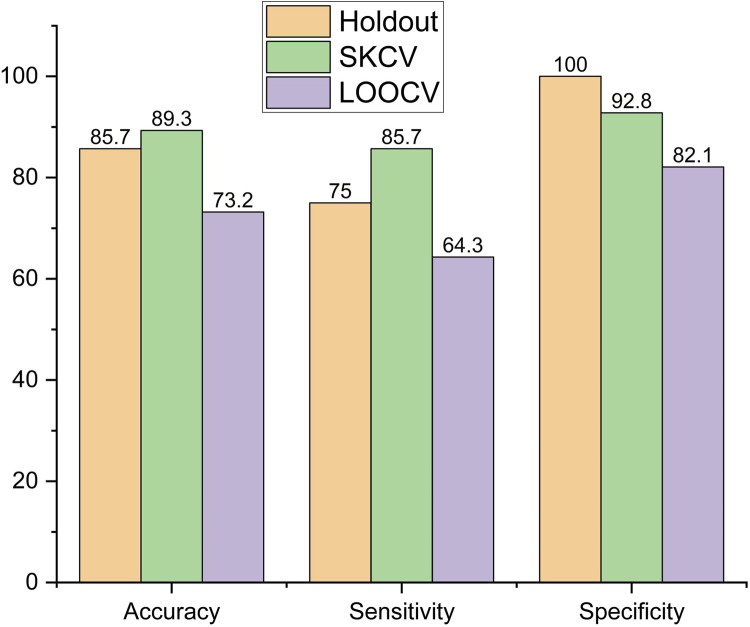
Performance metric of decision tree.

**FIGURE 9:**
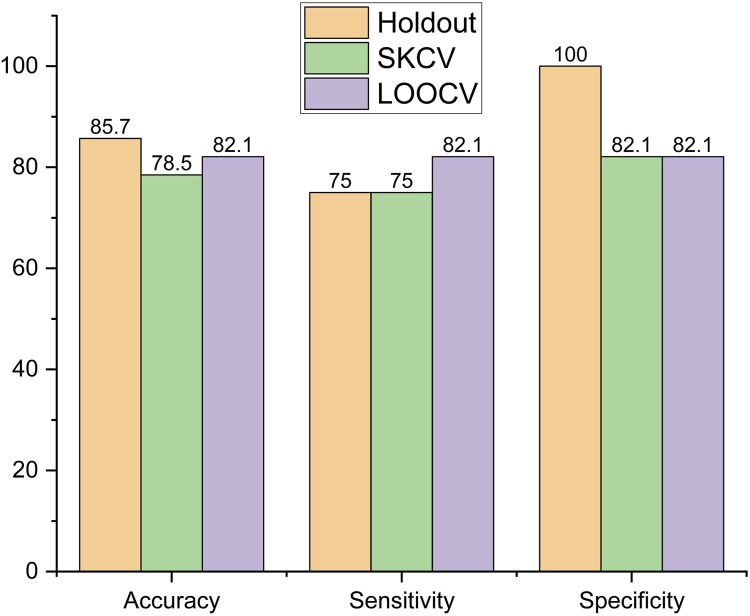
Performance metric of random forest.

The performance metrics of the models, viz., logistic regression, SVM, decision tree, and random forest, are shown in Figures 6–9, respectively.

It is observed that in logistic regression, as shown in Figure 6, accuracy remained the same when the sampling method was changed from holdout to SKCV and increased by 2.1% from 85.7% to 87.5% when it was changed to LOOCV. Sensitivity increased by 9.46% from 75% to 82.1% when the sampling method was changed from holdout to SKCV or LOOCV. Specificity, however, decreased by 10.8% and 7.2% from 100% to 89.8% and from 100% to 92.8%, respectively, when the sampling method was changed from holdout to SKCV and LOOCV, respectively. The specificity obtained by the LOOCV sample was 3.87% higher than that obtained using the SKCV sample.

In the SVM, accuracy increased by 4.45%, from 78.6% to 82.1%, when the sampling method was changed from holdout to SKCV, and decreased by 2.41%, from 78.6% to 76.7%, when the sampling method was changed from holdout to LOOCV. Sensitivity remained the same for holdout- and SKCV-sampled data, while it increased by 4.6%, from 75% to 78.5%, when LOOCV sampled data were employed. Specificity for the SKCV sampled data increased by 6.7% and 16.01% in comparison to that of the data sampled by the holdout and LOOCV methods, respectively, as shown in Figure 7.

In the decision tree model, accuracy was highest for the data sampled using SKCV, with a decrease of 4%, from 89.3% to 85.7%, for data sampled by holdout and a larger decrease of 18%, from 89.3% to 73.2%, for data sampled by LOOCV, as shown in Figure 8. The trend was also similar for sensitivity, which peaked at 85.7% for data sampled by SKCV but decreased by 12.48% for data sampled by holdout and LOOCV. Specificity is the highest for data sampled by the Holdout method but decreased by 7.2% and 17.9% for data sampled by SKCV and LOOCV, respectively.

In the random forest, accuracy was highest for the holdout-sampled data, with a decrease of 4.2%, from 85.7% to 78.5%, and 8.4%, from 85.7% to 82.1%, for data sampled by LOOCV and SKCV, respectively. Sensitivity is highest for the LOOCV-sampled data but decreased by 8.64%, from 82.1% to 75%, for both holdout- and SKCV-sampled data, as shown in Figure 9. Sensitivity is highest for the holdout-sampled data but decreased by 17.9%, from 100% to 82.1%, for both SKCV- and LOOCV-sampled data.

## 4 Muscular activity of the periarticular muscles in KOA and healthy subjects

The identification of KOA through pedobarographic variables and pelvis and trunk motion variables can be inferred from the above section. Effectively monitoring rehabilitation therapies for KOA using these variables necessitates establishing the impact of KOA on the strength of the periarticular muscles around the knee, which are part of the prime movers of knee flexion and extension (Levangie et al., 2011), and the rectus femoris and biceps femoris are part of the muscle group. Increased muscle force in the rectus femoris (Yagi et al., 2022) and increased muscle activation in the biceps femoris (Hortobágyi et al., 2005) for KOA subjects make it suitable for sEMG studies to differentiate between KOA and healthy subjects. In addition, sEMG features from the rectus femoris (Khader et al., 2024) and biceps femoris (Chen et al., 2019; Khader et al., 2024) have been previously employed in the identification of KOA. Thus, rectus femoris and biceps femoris are employed for the collection of sEMG data from healthy and KOA subjects, using them for the identification of KOA subjects. It is conducted by clustering analysis utilizing sEMG signals collected from the rectus femoris and biceps femoris caput longus bilaterally from two healthy subjects and six KOA subjects from the dataset. Prior to clustering, sEMG signals are filtered and analyzed to extract features in time, frequency, and time–frequency domains. The number of features is reduced using principal component analysis (PCA), which is then used for clustering analysis.

### 4.1 sEMG data collection

sEMG data of six subjects with varying degrees of KOA and two healthy subjects are collected to assess the muscle activity of the subjects during different activities. The various activities considered are standing under four different conditions for 60 s and walking. The standing conditions include walking at a self-selected speed and standing on firm ground with eyes open (Firm EO), firm ground with eyes closed (Firm EC), foam with eyes open (Foam EO), and foam with eyes closed (Foam EC). Table 7 provides us with a summary of the condition of the subjects along with the activities undertaken by each subject.

**TABLE 7:** Summary of the subjects’ condition during sEMG analysis.

Subject	Health condition	Activities undertaken
Subject 1	Healthy (H1)	Firm EO, Firm EC, Foam EO, Foam EC, and walking
Subject 2	Healthy (H2)	Firm EO, Firm EC, Foam EO, Foam EC, and walking
Subject 3	KOA Grade 3 (OA1_G3)	Firm EO, Firm EC, and Foam EO.
Subject 4	KOA Grade 1 (OA2_G1)	Firm EO, Foam EO, and Foam EC.
Subject 5	KOA Grade 2 (OA3_G2)	Firm EO, Firm EC, Foam EO, Foam EC, and walking
Subject 6	KOA Grade 1 (OA4_G1)	Firm EO, Firm EC, and walking
Subject 7	KOA Grade 1 (OA5_G1)	Walking
Subject 8	KOA Grade 1 (OA6_G1)	Walking

**FIGURE 10:**
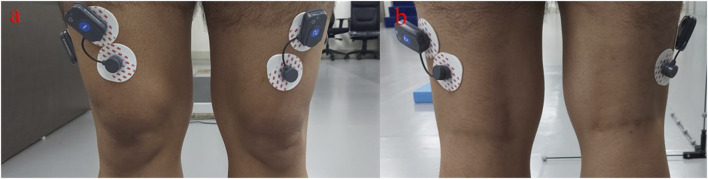
sEMG electrodes placed on **(A)** rectus femoris and **(B)** bicep femoris caput longus.

Data collection is done using wireless sEMG sensors from BTS Bioengineering, as shown in Figure 10. The muscles considered are the bilateral rectus femoris and biceps femoris caput longus, which are part of the quadriceps and hamstring group of muscles.

### 4.2 Feature selection using time-series, frequency, and time–frequency analysis

Along with sEMG signals, various noises and movement artifacts are also detected, so the required information remains together with the raw sEMG signal. It is thus difficult to assess the subjects using raw signals. Thus, sEMG features from different domains, which include time, frequency, and time–frequency domain features, are to be employed for the assessment (Chowdhury et al., 2013). Time domain features are considered as they have been reported to successfully classify healthy and knee-pathological subjects (Naik et al., 2018). The time domain features extracted here include “the Hudgins’ features,” i.e., the mean absolute value (MAV), MAV slope, slope sign changes (SSC), waveform length (WL), and zero crossings (ZCs) (Hudgins et al., 1993). In addition to that, average amplitude change (AAC), difference absolute standard deviation value (DASDV), integrated EMG (iEMG), kurtosis, log, root mean square (RMS) value, variance (var), and skewness are also evaluated in the time domain. Thus, 13 features are considered in the time domain. Frequency domain features have been established to be the best for assessing muscle fatigue (Cifrek et al., 2009). A time-domain EMG signal is transformed to the frequency domain using periodogram analysis, where the square of the absolute value of the Fourier transform of the EMG signal is divided by the signal length (Phinyomark et al., 2012). Thus, five frequency features, namely, the mean frequency (MNF), median frequency (MDF), mean power (MNP), and total power (TTP), are extracted from the EMG signal. MNF is the average frequency of the power spectrum of the EMG signal. MDF is the frequency at which the EMG power spectrum is divided into two regions with equal amplitudes. TTP is the aggregate of the EMG power spectrum and is also known as energy and the zero spectral moment. TTP and MNP are the frequency domain features that extract the same information as time domain features such as iEMG, RMS, and MAV based on the energy information as muscle fatigue results in an increase in EMG signal amplitude (Phinyomark et al., 2012). In addition to time and frequency analyses, time–frequency analysis is performed using the continuous wavelet transform (CWT) as it has been reported to effectively document quadriceps fatigue during knee extension exercise (So et al., 2009). In addition, wavelet neural network models using sEMG have been employed to estimate knee joint angles (Li et al., 2020). In CWT, one of the crucial steps is the selection of the “mother wavelet,” which depends on the study application. The 5th order of coiflet is reported to provide the perfect reconstruction of the sEMG signal. Furthermore, symlet4 and symlet5 have been employed to determine muscle failure. Daubechies’s functions (db2, db4, db6, db44, and db45) have been reported to be successfully applied for analyzing sEMG signals (Chowdhury et al., 2013). Thus, we have employed coiflet5, symlet4, symlet5, db2, and db4 as mother wavelets for the analysis of the sEMG signals and obtained five features, namely, the zero crossing rate (ZRC), root mean square (RMS), maximum amplitude, phase duration, and number of peaks for each signal.

### 4.3 Cluster analysis

Using the features obtained from each analysis, cluster analysis is performed in each experimental condition, namely, Firm EO, Firm EC, Foam EO, Foam EC, and walking. Using the time-series analysis, each muscle is found to have 13 features. As data are collected from four muscles, a total of 13 × 4, i.e., 52 features for each subject, are obtained in each experimental condition. The large number of features is reduced by employing PCA. The number of principal components (PCs) extracted is based on the condition that the maximum variance is explained by the least number of PCs. The 52 features are reduced to 5, 4, 4, 3, and 5 PCs for Firm EO, Firm EC, Foam EO, Foam EC, and walking condition, respectively. Using PCs, the K-nearest neighbor (KNN) algorithm is employed unsupervised to divide the subjects into clusters. Table 8 shows the clustering pattern obtained under the different experimental conditions for time-series features. It can be seen that PCs from time-series features were able to cluster the subjects accurately only for the Foam EC condition, with two healthy subjects and two OA subjects, one with Grade 1 and the Grade 2 severity. In the Firm EC condition, which contains five subjects, the OA subjects with Grade 1 and Grade 2 severity were clustered together with healthy subjects, while the OA subjects with Grade 3 severity were clustered separately. In the Firm EO condition, six subjects (two healthy and four OA) are considered. Two healthy subjects and two OA subjects with Grade 1 and Grade 2 severity are clustered together, and two OA subjects with Grade 1 and Grade 3 severity are then clustered separately. In the FOAM EO condition, five subjects (two healthy and three OA) are considered. One healthy and one OA Grade 2 subject are clustered together, and one healthy subject and two OA subjects with Grade 1 and Grade 3 are clustered together separately. In the walking condition, six subjects (two healthy and four OA subjects) are considered. Two healthy and two OA subjects with Grade 1 are clustered together, and two OA subjects, one with Grade 1 and one with Grade 2, are clustered together separately.

**TABLE 8:** Cluster analysis on the time-series features.

	Subject name	Subject 1	Subject 2	Subject 3	Subject 4	Subject 5	Subject 6	Subject 7	Subject 8
	Actual condition	H1	H2	OA1_G3	OA2_G1	OA3_G2	OA4_G1	OA5_G1	OA6_G1
Cluster assigned with data from different experimental conditions	Firm EO	1	1	0	0	1	1	-	-
	Firm EC	1	1	0	—	1	1	-	-
	Foam EO	1	0	0	0	1	-	-	-
	Foam EC	0	0	—	1	1	-	-	-
	Walking	1	1	—	—	0	1	1	0

Frequency analysis provides five features for each muscle. Thus, a total of 5 × 4, i.e., 20 features for each subject, is obtained in each experimental condition. In each case, PCA is conducted to reduce the number of features. PCA reduces the 20 features to 4, 4, 4, 3, and 4 PCs for Firm EO, Firm EC, Foam EO, Foam EC, and walking condition, respectively. Using the PCs, the KNN algorithm is employed unsupervised to divide the subjects into clusters. Table 9 shows the clustering pattern obtained under the different experimental conditions for the frequency features. The PCs from the frequency features also cluster the subjects similarly, with the accurate cluster available with Foam EC condition. The clustering behavior for the other experimental conditions is similar to that of the time-series features except for the Foam EO condition, where the healthy subjects and the OA subject with Grade 1 severity are clustered together, and the OA subjects with Grade 2 and Grade 3 severity are clustered together separately.

**TABLE 9:** Cluster analysis on the frequency features.

	Subject name	Subject 1	Subject 2	Subject 3	Subject 4	Subject 5	Subject 6	Subject 7	Subject 8
	Actual condition	H1	H2	OA1_G3	OA2_G1	OA3_G2	OA4_G1	OA5_G1	OA6_G1
Cluster assigned with data from different experimental conditions	Firm EO	1	1	0	0	1	1	—	—
	Firm EC	1	1	0	—	1	1	—	—
	Foam EO	1	1	0	1	0	—	—	—
	Foam EC	0	0	—	1	1	—	—	—
	Walking	1	1	—	—	0	1	1	0

Similarly, wavelet analysis, i.e., time–frequency analysis, also provides us with five features for each muscle. Thus, we have a total of 5 × 4, i.e., 20 features for each subject in each experimental condition. In each case, at first, PCA is conducted to reduce the number of features. PCA reduces the 20 features to 5, 4, 3, 3, and 5 PCs for Firm EO, Firm EC, Foam EO, Foam EC, and walking condition, respectively, with coif5 as the mother wavelet. In the case of the db2 mother wavelet, PCA reduces the 20 features to 5, 4, 4, 3, and 5 PCs for Firm EO, Firm EC, Foam EO, Foam EC, and walking condition, respectively. Considering the db4 mother wavelet, PCA reduces the 20 features to 5, 4, 4, 3, and 5 PCs for Firm EO, Firm EC, Foam EO, Foam EC, and walking condition, respectively. With the sym4 mother wavelet, PCA reduces the 20 features to 5, 4, 4, 3, and 5 PCs for Firm EO, Firm EC, Foam EO, Foam EC, and walking condition, respectively. With the sym5 mother wavelet, PCA reduces the 20 features to 5, 4, 4, 3, and 5 PCs for Firm EO, Firm EC, Foam EO, Foam EC, and walking condition, respectively. Using PCs, the KNN algorithm is employed unsupervised to divide the subjects into clusters. Table 10 shows the clustering pattern obtained under the different experimental conditions using wavelet features. The clustering behavior of the PCs obtained from wavelet features is the same, irrespective of the choice of the mother wavelet. Accurate clustering is obtained for Firm EO, Foam EO, and Foam EC conditions. In the Firm EO condition, six subjects (two healthy and four OA) are considered. The healthy subjects are clustered together, and the four OA subjects with varying severity are clustered together separately. In the Firm EO condition, five subjects (two healthy and three OA) are considered. The healthy subjects are clustered together, and the three OA subjects with varying severity are clustered together separately. In the Foam EC condition, four subjects (two healthy and two OA) are considered. The healthy subjects are clustered together, and two OA subjects with varying severity are clustered together separately. In the case of Firm EC condition, five subjects (two healthy and three OA) are considered. One healthy subject is clustered together, and the other subjects (one healthy and three OA) are clustered together separately. In the walking condition, six subjects (two healthy and four OA) are considered. Two healthy subjects, one OA subject with Grade 2 severity and one OA subject with Grade 1 severity, are clustered together; and two OA subjects with Grade 1 are clustered together separately.

**TABLE 10:** Cluster analysis on the wavelet features.

	Subject name		Subject 1	Subject 2	Subject 3	Subject 4	Subject 5	Subject 6	Subject 7	Subject 8
	Actual condition		H1	H2	OA1_G3	OA2_G1	OA3_G2	OA4_G1	OA5_G1	OA6_G1
Cluster assigned with data from different experimental conditions	Firm EO	coif5	1	1	0	0	0	0	—	-
		db2	1	1	0	0	0	0	—	-
		db4	1	1	0	0	0	0	—	-
		sym4	1	1	0	0	0	0	—	-
		sym5	1	1	0	0	0	0	—	-
	Firm EC	coif5	0	1	1	—	1	1	—	-
		db2	0	1	1	—	1	1	—	-
		db4	0	1	1	—	1	1	—	-
		sym4	0	1	1	—	1	1	—	-
		sym5	0	1	1	—	1	1	—	-
	Foam EO	coif5	1	1	0	0	0	—	—	-
		db2	1	1	0	0	0	—	—	-
		db4	1	1	0	0	0	—	—	-
		sym4	1	1	0	0	0	—	—	-
		sym5	1	1	0	0	0	—	—	-
	Foam EC	coif5	0	0	—	1	1	—	—	-
		db2	0	0	—	1	1	—	—	-
		db4	0	0	—	1	1	—	—	-
		sym4	0	0	—	1	1	—	—	-
		sym5	0	0	—	1	1	—	—	-
	Walking	coif5	1	1	—	—	1	0	1	0
		db2	1	1	—	—	1	0	1	0
		db4	1	1	—	—	1	0	1	0
		sym4	1	1	—	—	1	0	1	0
		sym5	1	1	—	—	1	0	1	0

## 5 Discussion

With a worldwide high prevalence, KOA has significantly affected the quality of life of a large population. Because of its degenerative nature, early identification and, thus, early intervention greatly affect the management of KOA. The current gold standard to identify KOA is X-ray imaging, which is best suited for assessing the progression of the disorder, and MRI is more effective in ruling out OA but is expensive. In addition, there is a risk of radiation exposure in the case of X-rays. Hence, it is not effective for continuous monitoring of any rehabilitation regimens. The interdependency of the kinetic and kinematic variables of human movement with the muscle activity of the associated joints provides an alternate, non-invasive knee health monitoring technique. It is conducted by monitoring the pedobarographic data and pelvis and trunk motion as they are reported to be affected by the condition of the muscles around the knee (Gajdosik et al., 1994; Harty et al., 2005; Lee et al., 2020), which are in turn reported to be provided as physical therapy for the management of KOA (Bhatia et al., 2013).

Point-biserial correlation gives us 24 statistically significant features that are affected by KOA. Age is the most positively correlated variable, which is because of the nature of the disease, i.e., a chronic degenerative disorder (Anderson and Loeser, 2010). The TUG variables, which include one feature from the trunk F/E during sit-to-stand and stand-to-sit conditions, and the TUG time are the next most positively correlated variables. This is because KOA has been reported to affect both gait and gaze during TUG (Rossignol et al., 2023). The positive correlation between the double-stance phase % and the stance phase (right and left) % is probably because of the impaired balance control due to KOA (Kim et al., 2011). The increased double-stance phase also results in a reduction of the swing phase and single-limb support line. BMI is found to be positively correlated with KOA because obesity has been associated with high risks of KOA (Zheng and Chen, 2015). Heel pressure is negatively correlated with KOA, thus inferring low heel pressure in KOA subjects. It is because of the insufficient knee extension during the heel-contact phase (Saito et al., 2013). A negative correlation is also observed for the spatiotemporal variables such as step length, stride length, and effective velocity, which are indicative of the abnormal knee joint loading adaptations due to the KOA (Chen et al., 2003). The SD of pelvis rotation on both the right and left sides is found to be negatively correlated with KOA, which suggests reduced variation in the pelvis rotation due to KOA. This is because there is reduced pelvic rotation in KOA subjects (Tanaka et al., 2007; Van Der Esch et al., 2011), which is a compensatory change adapted to minimize the load on the affected knee (Van Der Esch et al., 2011). Thus, pelvic rotation exercises can be part of the rehabilitation regimen targeted to address KOA (Tanaka et al., 2007). Age and SD of trunk F/E (sit to stand) are the most positively affected variables due to KOA, and velocity is the most negatively affected variable due to KOA.

ML models, namely, logistic regression, SVM, decision tree, and random forest, have been successful in classifying KOA with the highest accuracy of 89.3% and the highest sensitivity of 85.7% with decision tree as a classifier and SKCV as the data sampling method. A specificity of 100% is obtained for three classifiers, namely, logistic regression, decision tree, and random forest with holdout as the sampling method. However, SKCV or LOOCV data sampling methods provide more robust results and are more adaptive to overfitting problems. Not considering the holdout sampling method, the specificity is the highest at 92.8% using decision tree as a classifier and SKCV as the data sampling method. The best performance is obtained with SKCV-sampled data because it ensures that each fold maintains the same distribution as the original dataset. This helps the model learn equally in all the folds and makes it sensitive to imbalances in the dataset. In addition, decision tree excels at extracting meaningful interactions between features, especially if they are non-linear in nature, compared to logistic regression and SVM. The better performance of decision tree in comparison to random forest may seem counterintuitive as random forest is an ensemble method that builds multiple decision trees; however, it may be because decision trees can capture interactions between features at a finer level compared to random forest and in the absence of excessive noise in the data, it may lead to better-performing decision trees. Thus, decision tree is the best-performing algorithm for the identification of subjects with KOA using pedobarographic data and pelvis and trunk motion. This reaffirms the possibility of the identification of KOA using variables from joints other than the knee, which was also reported by Kobsar et al. (2017), in which KOA rehabilitation responses were classified according to their effectiveness using wearable sensors in the back, thigh, and shank.

KOA greatly affects the muscle activity of the rectus femoris and biceps femoris caput longus. PCs from time-series features can distinguish between Grades 1 and 2 of KOA and healthy subjects during the Foam EC standing condition. They can also distinguish between Grade 3 KOA and other grades of KOA and healthy subjects during the Firm EC condition. They, thus, can distinguish between the early stages of KOA and healthy subjects. PCs from time-series features are not effective in distinguishing between KOA subjects and healthy subjects during Firm EO and Foam EO standing conditions and walking. In addition to Foam EC and Firm EC conditions, PCs from the frequency features are also able to distinguish between KOA with Grade 2 or higher severity and healthy subjects. It may be due to the fact that frequency domain features are sensitive to the effect of muscle fatigue (Cifrek et al., 2009) during any activity and hence are able to detect the strain on muscles due to KOA of Grade 2. However, Grade 1 KOA and healthy subjects are indistinguishable by this feature. PCs from the wavelet features performed the best in distinguishing between KOA subjects of different grades and healthy subjects for Firm EO, Foam EO, and Foam EC, by perfect distinction. It may be because time–frequency domain analysis provides a deeper understanding of the electrophysiological processes behind the neuromuscular activations (Di Nardo et al., 2022), and continuous wavelet transforms have also been reported to outperform other time–frequency analyses for both simulated and real EMG recordings (Karlsson and Gerdle, 2001). However, muscle activity from the rectus femoris and biceps femoris caput longus during walking is not found to distinguish between KOA subjects and healthy subjects. This is because during walking, balance, support, and progression are mostly contributed by five muscle groups, namely, the gluteus maximus, gluteus medius, vasti, gastrocnemius, and soleus (Lim et al., 2022).

Thus, KOA is diagnosed using gait variables for joints other than the knee, such as pedobarographic data and pelvis and trunk motion, along with the comparison of muscular activity in the bilateral rectus femoris and biceps femoris caput longus muscles for a section of the KOA and healthy subjects. Data are collected using wearable sensors, except for pedobarographic data, which can also be collected using wearable flexible insoles (Stassi et al., 2013). This allows for the identification of KOA through variables that can be captured using wearable sensors in real-world scenarios. Moreover, the establishment of the effect on muscular activity of knee periarticular muscles due to KOA through clustering analysis shows the complementary relationship between the gait variables and sEMG data. This opens up the possibility of monitoring hamstring and quadriceps strengthening as part of rehabilitation therapy to address KOA. The effect of KOA on the periarticular muscles of the knee is also reported by Ghazwan et al. (2022).

## 6 Limitations

A limitation of this study is that the EMG analysis was performed on only a few subjects and sEMG data were collected only for two major muscles responsible for knee joint movement. The periarticular muscles surrounding the knees contribute majorly during standing; however, the major muscles contributing during other activities of daily life are from different groups. Thus, the inclusion of at least one muscle from each of the major muscle groups in the lower limb will encompass and translate the study into more activities and also help us consider the synergistic behavior of the muscles.

## 7 Conclusion

This study establishes the capability of detecting KOA using gait variables from joints other than the knee. It employs pedobarographic data and pelvis and trunk ROM for the analysis. This offers a non-invasive and accessible method for the detection of KOA. The variables most affected by KOA are the SD of trunk F/E (sit to stand) and velocity. In addition, the study associated KOA with reduced pelvic rotation and thus suggests pelvis rotation exercises as part of a rehabilitation regimen targeted to address the effects of KOA. Furthermore, evidence of altered muscle activity in the rectus femoris and biceps femoris caput longus, which are part of the quadriceps and hamstring group of muscles, is found in subjects affected by KOA through cluster analysis. Thus, it can be inferred that KOA affects both mobility and muscle condition simultaneously, and both datasets complement each other. Hence, gait data can be employed to identify KOA subjects and perform a preliminary assessment and monitoring approach to gauge the effectiveness of rehabilitation therapies aimed at addressing KOA through muscle strengthening. Given the feasibility of collecting pedobarographic data and pelvis and trunk motion using wearable sensors with minimal sample preparation and its non-radiographic nature, the proposed method can be seamlessly integrated not only in a laboratory setting but also in real-world environments. Different combinations of machine learning models and data sampling methods have been employed to understand this behavior, and the decision tree with data sampled using SKCV is found to be the best classifier of KOA using gait data. The activities to be considered for monitoring the assessment include walking and standing under different conditions, such as Firm EO, Foam EO, and Foam EC.

## Data Availability

The original contributions presented in the study and the data used for classification in the study are included in the article/Supplementary Material; further inquiries can be directed to the corresponding author.
